# Transient Decrease of Circulating and Tissular Dendritic Cells in Patients With Mycobacterial Disease and With Partial Dominant IFNγR1 Deficiency

**DOI:** 10.3389/fimmu.2020.01161

**Published:** 2020-06-26

**Authors:** Laura Dotta, Donatella Vairo, Mauro Giacomelli, Daniele Moratto, Nicola Tamassia, William Vermi, Silvia Lonardi, Jean-Laurent Casanova, Jacinta Bustamante, Silvia Giliani, Raffaele Badolato

**Affiliations:** ^1^Department of Pediatrics, A. Nocivelli Institute for Molecular Medicine, ASST Spedali Civili of Brescia, Brescia, Italy; ^2^Department of Molecular and Translational Medicine, A. Nocivelli Institute for Molecular Medicine, University of Brescia, Brescia, Italy; ^3^A. Nocivelli Institute for Molecular Medicine, ASST Spedali Civili of Brescia, Brescia, Italy; ^4^Section of General Pathology, Department of Medicine, University of Verona, Verona, Italy; ^5^Department of Molecular and Translational Medicine, University of Brescia, Brescia, Italy; ^6^Laboratory of Human Genetics of Infectious Diseases, Necker Branch, INSERM UMR 1163, Paris, France; ^7^University of Paris, Imagine Institute, Paris, France; ^8^St. Giles Laboratory of Human Genetics of Infectious Diseases, Rockefeller Branch, The Rockefeller University, New York, NY, United States; ^9^Pediatric Hematology and Immunology Unit, Necker Hospital for Sick Children, AP-HP, Paris, France; ^10^Howard Hughes Medical Institute, New York, NY, United States; ^11^Center for the Study of Primary Immunodeficiencies, Necker Hospital for Sick Children, AP-HP, Paris, France; ^12^Department of Clinical and Experimental Sciences, Department of Pediatrics, A. Nocivelli Institute for Molecular Medicine, University of Brescia, Brescia, Italy

**Keywords:** partial IFNγR1 deficiency, IFNγ, dendritic cells deficiency, mycobacteria, MSMD

## Abstract

Interferon-γ receptor 1 (IFNγR1) deficiency is one of the inborn errors of IFN-γ immunity underlying Mendelian Susceptibility to Mycobacterial Disease (MSMD). This molecular circuit plays a crucial role in regulating the interaction between dendritic cells (DCs) and T lymphocytes, thus affecting DCs activation, maturation, and priming of T cells involved in the immune response against intracellular pathogens. We studied a girl who developed at the age of 2.5 years a *Mycobacterium avium* infection characterized by disseminated necrotizing granulomatous lymphadenitis, and we compared her findings with other patients with the same genetic condition. The patient carried a heterozygous 818del4 mutation in the *IFNGR1* gene responsible of autosomal dominant (AD) partial IFNγR1 deficiency. During the acute infection blood cells immunophenotyping showed a marked reduction in DCs counts, including both myeloid (mDCs) and plasmacytoid (pDCs) subsets, that reversed after successful prolonged antimicrobial therapy. Histology of her abdomen lymph node revealed a profound depletion of tissue pDCs, as compared to other age-matched granulomatous lymphadenitis of mycobacterial origin. Circulating DCs depletion was also observed in another patient with AD partial IFNγR1 deficiency during mycobacterial infection. To conclude, AD partial IFNγR1 deficiency can be associated with a transient decrease in both circulating and tissular DCs during acute mycobacterial infection, suggesting that DCs counts monitoring might constitute a useful marker of treatment response.

## Introduction

Interferon-γ receptor 1 (IFNγR1) deficiency was discovered in 1996 as the first genetic etiology of the Mendelian Susceptibility to Mycobacterial Disease (MSMD) (1, 2). MSMD is a rare inherited primary immunodeficiency due to impaired IFN-γ-mediated immunity that leads to selective predisposition to clinical disease caused by weakly virulent mycobacteria, such as bacillus Calmette-Guerin (BCG) vaccines and non-tuberculous environmental mycobacteria (EM) (3). This condition also exposes to higher risk of infection from the more virulent *Mycobacterium tuberculosis, Candida* spp., *Salmonella*, and, more rarely, to other intra-macrophagic bacteria, fungi, or parasites. Mutations in the *IFNGR1* gene account for the altered cell surface expression of the high affinity ligand-binding chain of the receptor and impair the cellular response to IFNγ. *IFNGR1* mutations result in five distinct clinical phenotypes depending on the pattern of inheritance (dominant or recessive), the impact of the mutation (complete or partial defect) and the expression of the mutant allele. Patients with autosomal recessive (AR) complete IFNγR1 deficiency display the most severe clinical phenotype with life-threatening disseminated infections starting in early childhood (3). In this form, the majority are null mutations that prevent the production of the receptor; alternatively, in-frame deletions and missense mutations are reported to cause the production of IFNγR1 that, altered in its extracellular domain, in unable to bind the ligand (3). It has been hypothesized that high levels of serum IFNγ may be responsible for the high rate of graft rejection observed in these patients after hematopoietic stem cell transplantation (HSCT), that currently represents the only curable treatment (4). In patients with AR partial IFNγR1 deficiency, homozygous mutations include specifically the I87T, V63G, and M1K mutations: in leukocytes the receptor is expressed but not responsive to high concentrations of IFNγ. The clinical outcome of infections and the survival are favorable (5). Finally, the autosomal dominant (AD) form of partial IFNγR1 deficiency includes heterozygous mutations in the cytoplasmic domain of the receptor leading to truncated molecule that accumulates at the cell surface, binds IFNγ, but cannot transduce the signal. Typically, these patients manifest mycobacterial disease at an older age (late childhood/adulthood) and are successfully treated with prolonged antimicrobial treatment (6, 7). The susceptibility to mycobacteria and to other intracellular opportunistic pathogens is also common to autosomal defects in *IFNGR2, IL12B, IL12RB1, STAT1, ISG15, IRF8* genes, or X-linked mutations in *NEMO* and *CYBB* genes, or in the recently described signal peptide peptidase-like 2 A (*SPPL2A*)*, IL12RB2*, and *IL23R* deficiencies, *TYK2* deficiency, or syndromic forms due to *ROR*γ*/ROR*γ*T* and *JAK1* deficiencies (3, 8–10). We herein report the immunological study of a pediatric patient with AD partial IFNγR1 deficiency who onset with disseminated mycobacterial disease that associated with defective dendritic cells (DCs) counts.

## Materials and Methods

### H&E and Immunohistochemistry

Four μm tissue sections from formalin-fixed and paraffin-embedded lymph nodes from the patient (PT) and two age-matched children were used for Hematoxylin and Eosin staining (H&E) and immunohistochemical staining to CD3 (clone LN10, Leica Biosystems), CD20 (clone L26, Leica Biosystems), and CD303 (clone 124B3.13, Dendritics) antigens. Briefly, after appropriate antigen retrival, antibodies were revealed using Novolink Polymer (Leica Biosystems) followed by Diaminobenzidine (DAB) and counterstained with hematoxylin.

### Sequencing of the *IFNGR1* Gene

Informed consent for hematological, immunological, and genetic tests was obtained from the patient's parents. The investigation protocol was approved by the local ethic committee of the Asst Spedali Civili of Brescia. We adhered to standard biosecurity and institutional safety procedures. The *IFNGR1* gene was analyzed by Sanger sequencing by using standard techniques. DNA was isolated from whole blood using QIAamp DNA Blood Mini Kit (Qiagen). *IFNGR1* gene was amplified by PCR and products were sequenced using BigDye Terminator Kit (Applied Biosystems). Sequences were analyzed with 310 Genetic Analyzer (Applied Biosystems).

### Expression of IFNγR1 and Phosphorylation of STAT1 on Monocytes by Flow Cytometry Assay

IFNγR1 cell-surface expression was assessed on monocytes (CD14+ cells) by using a standard flow cytometry protocol that requested a staining of 100 μl of fresh whole blood with PE-conjugated anti-human IFNγR1 mAb (BD Biosciencies). Blood samples were acquired using a FACSCalibur flow cytometer (Becton Dickinson, San Diego, Calif) and analyzed by the FlowJo software version 7.5 Software (TreeStar, Ashland, Ore). Cells were fixed and permeabilized, following the manufacturer's instructions (BD Biosciences). Similarly, analysis of STAT1 phosphorylation was performed on 100 μl of fresh whole blood that was left unstimulated or stimulated with IFN-γ (1,000 U/ml for 30 min). Blood cells were fixed and permeabilized, following the manufacturer's instructions (Becton Dickinson) and intracellular staining was performed using a PE-conjugated anti-human pSTAT1-Tyr-701 mAb (BD Pharmigen). Blood samples were acquired using a FACSCalibur flow cytometer and analysis of monocytes (CD14+ cells) pSTAT1 expression performed by FlowJo software.

### Cytokine Detection

Peripheral blood mononuclear cells (PBMCs) were obtained from EDTA-treated whole blood of the patient and healthy control by the standard density centrifugation procedure, in Ficoll Hypaque. Cytokine production was measured in the supernatants after 24 h of incubation by ELISA using matched paired Abs specific for IFNα (Mabtech) and CXCL10 (R&D systems).

### Immunophenotype Analysis by Flow Cytometry

Whole blood (100 or 200 ml) was stained for immunophenotypic analysis using a combination of monoclonal antibodies (mAb) (Becton Dickinson, San Diego, Calif), according to standard multiparametric flow cytometry protocols, in order to identify different lymphocytes and DCs subsets. Blood samples were acquired using a FACSCantoII flow cytometer (Becton Dickinson) and analyses were performed with the FlowJo software version 8.8.7 (TreeStar, Ashland, Ore). Immunological data were compared to the reference values of the “A. Nocivelli” Institute for Molecular Medicine of Brescia that performed the analysis, based on a database obtained from a pool of age-matched healthy subjects.

### Statistical Analysis

Statistical significance of immunological studies was analyzed by non-parametric two-side Mann– Whitney *U*-test with 95% confidence bounds. For all analyses *p* < 0.05 was considered statistically significant. Statistical analyses were performed using GraphPad Prism Version 5.0 (GraphPad Software, San Diego, CA).

## Results

### Disseminated Mycobacterial Infection in Partial Dominant IFNγR1 Deficiency

A 2.5-year-old female (P1) was admitted to the hospital because of 3 weeks of daily fever (over 38°C) associated with fatigue, vomiting and diarrhea. She was born in Italy from Pakistani non-consanguineous parents; her past medical history was uneventful, and the family history was unremarkable. She had not received BCG vaccine, nor had never been to Pakistan. At admission, her blood tests revealed microcytic anemia (Hb 6.5 g/dl, MCV 60.9 fl, RDW 19.4%), raised C-reactive protein (CRP 125 mg/L, range <5 mg/L) and procalcitonin (9.2 ng/ml, range <0.1 ng/ml), leukocytosis (WBC 21,600 cells/mmc) with neutrophilia (ANC 17,170 cells/mmc). She had normal lymphocyte count (ALC 3,230 cells/mmc), and monocyte count at the lower range (AMC 230 cells/mmc). On physical examination, she had mild spleen enlargement. The abdomen ultrasound and the computed tomography-scan revealed a spleen length of 8 cm [mean of normal age-related spleen size from 6.8 to 7.5 cm (11)] with multiple dishomogenous areas, slight hepatomegaly, without focal lesions, and multiple mediastinal and mesenteric lymph nodes showing internal colliquation. The infectious disease screening included blood cultures, *Brucella, Rickettsia, Bartonella, Coxiella, Leishmania, Toxoplasma, Plasmodium, Herpesviruses, Epstein-Barr virus*, and HIV serology, all tested negative. The Mantoux tuberculin skin test read after 48 h resulted on induration of 5 mm, while the QuantiFERON-TB Gold tested negative. She underwent a laparoscopic biopsy of an abdomen lymph node (20 × 14 mm) that showed a necrotizing granulomatous lymphadenitis with focal colliquation and the *Mycobacterium avium* was detected by PCR assay. The patient was commenced with the association therapy of clarithromycin, ethambutol and rifabutin, the latter discontinued after 6 months because of an acute drug-related uveitis. The dual therapy with clarithromycin and ethambutol was continued for 18 months. The patient fully recovered. At onset, routine immunological tests revealed slightly elevated levels of serum immunoglobulin (Ig) M (IgG 1,220 mg/dl -range 462–1,710 mg/dl-, IgA 167 mg/dl -range 27–173 mg/dl-, IgM 465 mg/dl -range 62–257 mg/dl-), and IgE (575 range KU/L -range <140 KU/L-), normal neutrophil respiratory burst assay, and normal bone marrow aspirate. The analysis of the lymphocytes subsets displayed a prevalence of effector T and B cells, an increased proportion of activated HLA-DR+ T cells, and reduction of NK cell percentages (CD16+CD56+ 2.7%/90 cells/mmc, normal range 5–28.4%/100–1,000 cells/mmc) (Table 1).

**Table 1:** The immunophenotype of the patient P1 heterozygous for 818del4 *IFNGR1* mutation at the onset of the disease and during follow-up.

	**Onset**	**7-months therapy**	**15-months therapy**	**Off-therapy Follow-up**
Age (years)	2.4	2.9	3.8	5.1
**Monocytes**				
Cells/mmc	**230** (285–500)	**270** (285–500)	440 (285–500)	630 (285–500)
**Total lymphocytes**				
Cells/mmc	3,350 (1,700–6,900)	5,720 (1,700–6,900)	6,050 (1,700–6,900)	4,990 (1,100–5,900)
**T CD3+** **Lymphocytes**				
Cells/mmc	2,569 (900–4,500)	3,106 (900–4,500)	4,761 (900–4,500)	4,091 (700–4,200)
%	76.7 (58.1–78.6)	54.3 (58.1–78.6)	78.7 (58.1–78.6)	82 (59.1–80.9)
**CD3+CD4+**				
Cells/mmc	1,217 (500–2,400)	903 (500–2,400)	2,432 (500–2,400)	1,787 (300–2,000)
%	47.4 (27.3–48.5)	29.1 (27.3–48.5)	51.1 (27.3–48.5)	43.7 (24.9–51.1)
CD3+CD4+ HLA-DR+ %	**26.8** (1.4–17.6)	16.4 (1.4–17.6)	13.5 (1.4–17.6)	10.2 (1.4–13.3)
CD3+CD4+ CD45RA+CCR7+ %	61.8 (53.6–81.4)	62 (53.6–81.4)	48.4 (53.6–81.4)	66.9 (37.8–80.3)
CD3+CD4+ CD45RA-CCR7+ %	13 (12.1–24.5)	20.9 (12.1–24.5)	32.8 (12.1–24.5)	18.7 (9.9–41.1)
**CD3+CD8+**				
Cells/mmc	644 (300–1,600)	599 (300–1,600)	928 (300–1,600)	1,288 (300–1,800)
%	25.1 (15–32.7)	19.3 (15–32.7)	19.5 (15–32.7)	31.5 (13.8–31.2)
CD3+CD8+ HLA-DR+%	23.7 (2.1–52)	10.9 (2.1–52)	26.5 (2.1–52)	30.1 (2.3–23.4)
CD3+CD8+ CD45RA+CCR7+%	13.6 (23–85.1)	17.6 (23–85.1)	14.5 (23–85.1)	15.4 (20.3–78.2)
CD3+CD8+ CD45RA-CCR7+%	2.8 (0.2–7.6)	1.7 (0.2–7.6)	3.9 (0.2–7.6)	2.8 (1.7–13.3)
**B CD19+** **Lymphocytes**				
Cells/mmc	626 (200–2,100)	2,162 (200–2,100)	1,101 (200–2,100)	698 (200–1,600)
%	18.7 (9.8–28)	37.8 (9.8–28)	18.2 (9.8–28)	14 (8.6–26–3)
CD19+ IgD+CD21^hi^%	43.9 (40.6–57.9)	61.4 (40.6–57.9)	55.4 (40.6–57.9)	47.5 (37.1–70.2)
CD19+ IgD-CD27+CD21^hi^%	3.5 (1.8–10.5)	2.1 (1.8–10.5)	3.9 (1.8–10.5)	12.2 (2.4–19.8)
PC CD38^hi^CD27^hi^CD20-CD138+%	**5.59** (0.11–2.2)	0.32 (0.11–2.2)	**4.3** (0.11–2.2)	**3.7** (0.06–2.7)
**CD3-CD16+CD56+** **NK Cells**				
Cells/mmc	**90** (100–1,000)	417 (100–1,000)	115 (100–1,000)	104 (90–900)
%	**2.7** (5–28.4)	7.3 (5–28.4)	1.9 (5–28.4)	**2.1** (3.3–22.8)
**pDC (BDCA2+CD123+CD4+)**				
%	**0.04** (0.16–0.76)	**0.01** (0.16–0.76)	0.17 (0.16–0.76)	0.35 (0.16–0.76)
**mDC (CD1c+CD4+CD19-CD14-)**				
%	**0.05** (0.18–0.92)	**0.05** (0.18–0.92)	0.38 (0.18–0.92)	0.45 (0.18–0.92)

*The values out of range are marked in bold numbers*.

**Figure 1:**
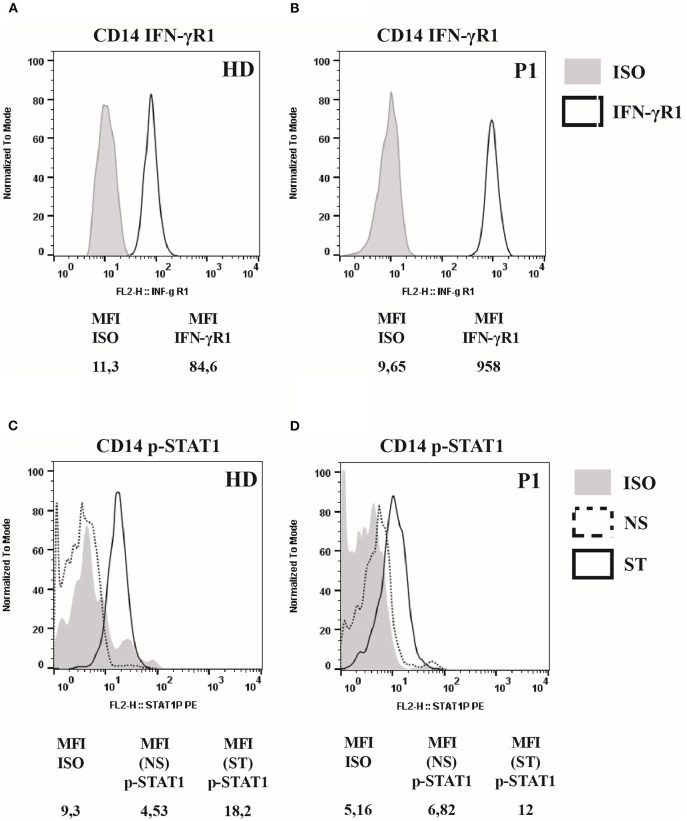
IFNγR1 cell-surface expression and activity in cells of patient heterozygous for 818del4 *IFNGR1* mutation. **(A)** The expression of IFNγR1 molecules (measured as Mean Fluorescence Intensity, MFI) at the surface of CD14-positive mononuclear blood cells, in the patient (P1) and a healthy donor (HD), by flow cytometry assay with a mouse antibody specific for human IFNγR1, and an isotype control. **(B)** The p-STAT1 level expression (measured as MFI), to analyze IFNγR1-mediated signaling, is detected by flow cytometry assay with a mouse antibody specific for human p-STAT1, and an isotype control, after incubation with IFN-γ (1,000 UI/ml) for 30 min, or medium alone, in CD14-positive mononuclear blood cells in the patient (P1) and a healthy donor (HD).

*IFNGR1* gene sequencing revealed a heterozygous small deletion in the exon 6, the c.819_822delTAAT (p.N274Hfs^*^2). This mutation is already reported as responsible of AD partial IFNγR1 deficiency (7). The flow cytometry analysis of IFNγR1 expression on patient's monocytes showed that the receptor was present at a higher level on cell's surface (Figures 1A,B), while STAT1 phosphorylation (pY-701) following stimulation with IFNγ was markedly reduced, as compared to the healthy control (Figures 1C,D).

### Study of DCs Counts Revealed a Depletion of Circulating and Tissular Subsets

**Figure 2:**
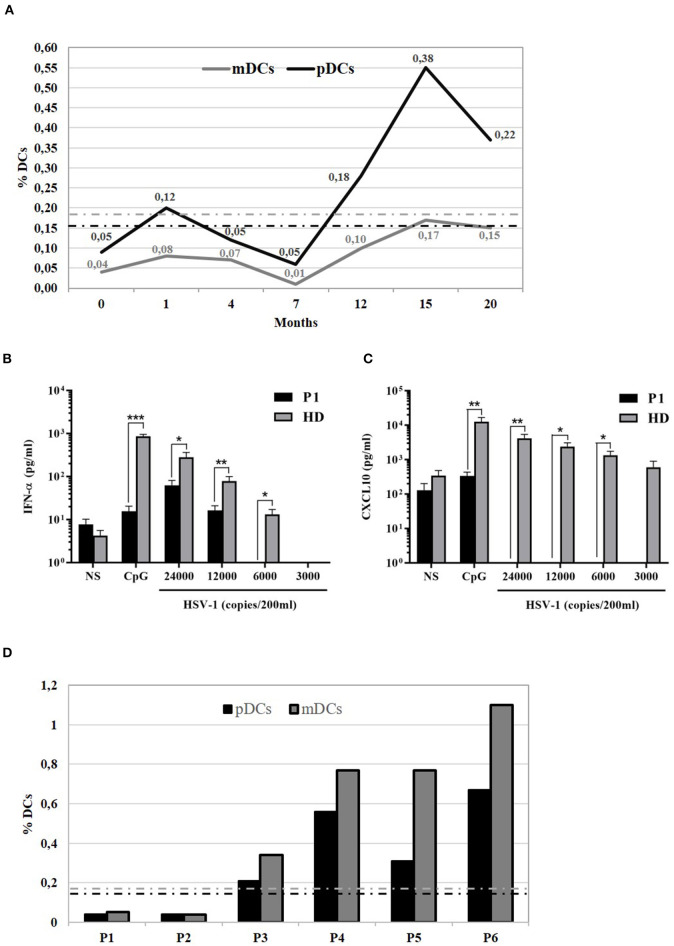
Functional analysis of 818del4 mutated IFNγR1 and study of patient's DCs. **(A)** Percentage of plasmacytoid dendritic cells (%pDCs) and myeloid dendritic cells (%mDCs) in P1 in timing (months) of therapy and follow-up (the dotted lines indicate the normal values, gray-colored for mDCs and black-colored for pDCs). IFN-α **(B)** and CXCL10 production **(C)** are measured in triplicate, by freshly isolated PBMCs after stimulation for 24 h with 6 mg/ml CpG, HSV-1 (copies/200 ml), or medium alone, in the patient (P1) and a healthy donor (HD). Statistical analysis by non-parametric test shows a significant difference (*p < 0.05, **p < 0.01, ***p < 0.001). **(D)** Plasmacytoid dendritic cells (%pDCs) and myeloid dendritic cells (%mDCs) are analyzed in other patients with mycobacterial infection: P2 has AD partial IFNγR1 deficiency and had acute mycobacterial infection during the evaluation of flow cytometry, while P3 has AD partial IFNγR1 deficiency but blood was collected when he was free of infection; P4, P5, and P6 presented a mycobacterial infection without any identified predisposing genetic cause (the dotted lines indicate the normal values, gray-colored for mDCs and black-colored for pDCs).

Analysis of DCs was performed based on specific cell surface markers. Myeloid/classical DCs (mDCs) express typical myeloid antigens (CD11c, CD13, CD33, CD11b) with the majority of them expressing CD1c/BDCA1, while plasmacytoid DCs (pDCs) express CD123, CD303/BDCA2, CD304/BDCA4, and produces IFNα after activation with CpG or viral infection (12–16). At the time of P1 mycobacterial infection diagnosis, we observed a marked reduction of pDCs equal to 0.04%/143 cells/mmc (normal range 0.16–0.76%) and of mDCs equal to 0.05%/179 cells/mmc (normal range 0.18–0.92%) (Table 1). Monitoring of DCs subsets during the infection treatment showed that the percentage of DCs progressively increased, reverting to the normal levels by the end of the treatment (18 months) and remaining normal in the following months (Figure 2A). In addition, ELISA measurements of IFNα and CXCL10 in 24-h supernatants of PBMCs incubated with CpG or with increasing copies of human Herpes Simplex Virus Type 1 (HSV1) demonstrated lower levels of these cytokines in P1 as compared to the healthy control (Figures 2B,C), which is in accord with the blood depletion of pDCs.

**Figure 3:**
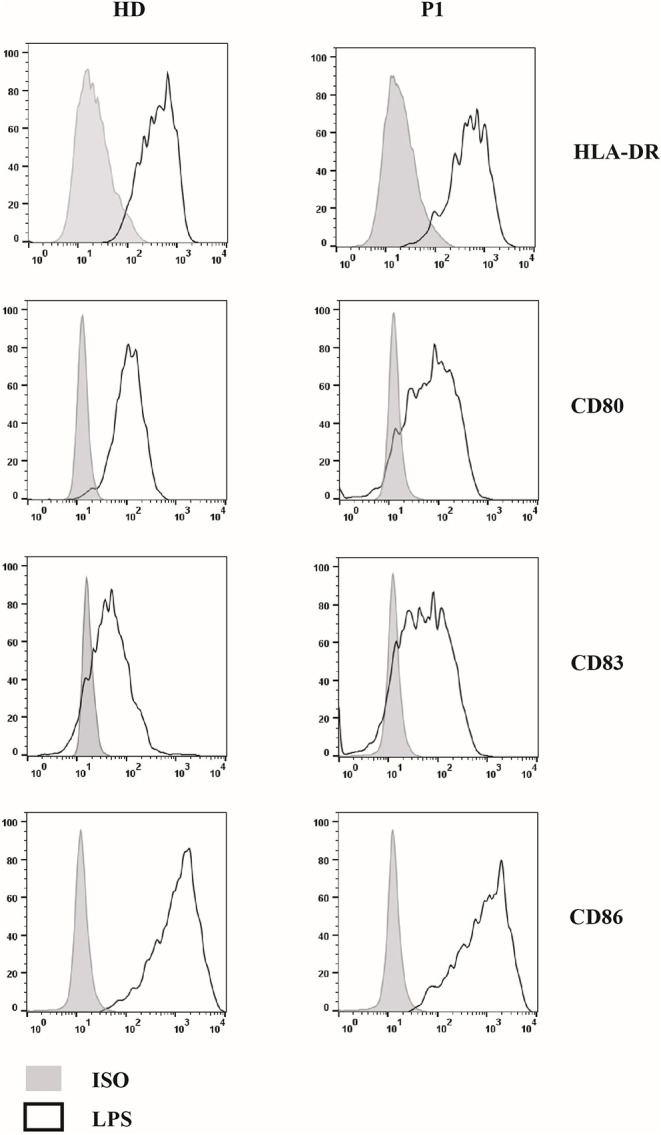
Study of 818del4 mutated DCs maturation pattern. DCs are cultured for 24 h with LPS for both the patient (P1) and a healthy donor (HD) and the expression of DCs maturation markers (HLA-DR, CD80, CD83, CD86) are analyzed by flow cytometry, together with an isotype control (PE- or PerCP- IgG): a similar pattern of maturation is present in both the patient and the healthy subject.

Next, we compared DCs subsets in other two patients (P2 and P3) with AD partial IFNγR1 deficiency, in P2 during a mycobacterial infection, while P3 was free of acute mycobacterial infection at the time the blood sample was collected. We also analyzed DCs in three other patients that had a mycobacterial cervical lymphadenopathy but still without any evidence of genetic mutations associated with MSMD. We detected a low number of both pDCs and mDCs subsets in P2, while DCs counts were normal in all the other investigated patients (Figure 2D). Finally, in P1, we studied DCs maturation by flow cytometry: the analysis of HLA-DR, CD80, CD83, and CD86 markers revealed that, after 24 h of *in vitro* culture with LPS, DCs exhibited a normal pattern of maturation (Figure 3).

### The Study of the Mycobacteria-Infected Lymph Node Biopsy Revealed pDCs Depletion

**Figure 4:**
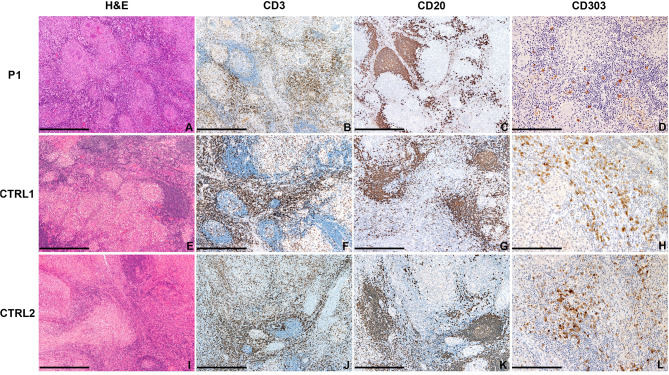
Abdominal lymph node histology. Sections are from lymph nodes of P1 **(A–D)** and two age-matched control **(E–L)** with mycobacterial infection, stained as labeled. On H&E, necrotizing granulomas are surrounded by lymphoid cells represented by CD20^+^ B-cells and CD3^+^ T-cells. CD303/BDCA2^+^ pDC are severely reduced as compared to matched controls. Original magnification: 40x (**A–C,E–G,I–K**; scale bar 500 μm), 100x (**D,H,L**; scale bar 200 μm).

In P1, the analysis of the lymph node biopsy revealed a granulomatous necrotizing lymphadenitis with BDCA2^+^ plasmacytoid dendritic cells reduction (Figures 4A–E), as compared with other age-matched granulomatous lymphadenitis of mycobacterial origin derived from control subjects (Figures 4F–L); the distribution of CD20^+^ B-cells and CD3^+^ T-cells of the nodal parenchyma was normal.

## Discussion

Immature DCs circulating in the peripheral blood can recognize and internalize pathogens for antigen presentation to T lymphocytes. Upon activation, DCs undergo maturation, secrete T cell activating cytokines such as IL-12, and migrate to lymph nodes where they have an essential role in orchestrating the host defense (11–15). In recent years, the identification of patients with DCs deficiency has allowed the investigation of the cellular pathways and genetic regulation of DCs haematopoiesis and their complex role in both innate and adaptive immunity. DCs deficiency (in both myeloid and plasmocytoid subsets) has been reported in patients with heterozygous germline *GATA2* mutations, a heterogeneous disorder characterized by familial myelodysplasia/acute myeloid leukemia (MSD/AML), the MonoMac (monocytopenia with *Mycobacterium avium* complex) syndrome, and/or the Emberger syndrome (primary lymphedema and MSD/AML) (17). In patients with Interferon Regulator Factor (IRF) eight mutations the AR variant causes a complete lack of circulating monocytes and DCs, while the AD variant associates with a selective depletion of CD11c+CD1c+ circulating dendritic cells (18, 19), as reported also in patients with the recently described SPPL2A (signal peptide peptidase-like 2A) deficiency, where a defective IL-12 and IL-23 production by mDCs may disrupt the priming of T lymphocytes (19, 20). Other disorders may display pancytopenia with monocyte and DCs deficiencies, from the WHIM (warts, hypogammaglobulinemia, infections, myelokathexis) syndrome (21), to defects in genes related to DCs development and functions, such as the CD40/CD40L deficiency, the MCH class II deficiency, the Wiskott-Aldrich syndrome, the Pitt-Hopkins Syndrome, or the IRF7 deficiency (22). The activation of DCs depends upon a crosstalk with CD4+ T lymphocytes, enabling the same DCs to prime cytotoxic T lymphocytes, polarize a Th1 T cells response, and regulate the immunity against intracellular pathogens (23–25). In lymph nodes, the T-cell response requires contact-dependent interaction with DCs, in order to activate cytokine production and cellular proliferation (26). Miro et al. investigated the T cell-driven maturation of DCs in a human *in vitro* model and supported how the secretion of IL-12 by DCs is dependent upon co-stimulatory stimuli, the CD40-CD40L-interaction during a direct cell-cell contact with CD4+ T cells, and the IFNγ production by T cells, in the presence of the antigen (27). Meanwhile, DCs maturation and activation requires T cell response to IL-12 and IFNγ: defect in the IL12β1R receptor prevents IL-12-mediated signaling and impairs both the expression of DCs maturation markers and the DCs production of IL-12. Moreover, the IFNγ receptor response in T cells is required for a functional interaction with DCs, likely through the STAT1-dependent IL-12R β2 expression on T cells via IFNγ signaling (28). Herein, we report the case of a *de novo* AD partial IFNγR1 deficiency that onset in early childhood as disseminated lymphadenitis due to *Mycobacterium avium*, well-responding to a treatment with clarithromycin and ethambutol. The acute phase of the mycobacterial infection associated with a marked reduction of both mDCs and pDCs, as measured in the peripheral blood. In addition, lymph node biopsy analysis demonstrated a profound decrease of pDCs also in the lymphoid tissue. It has been reported that DCs are decreased in HIV (29), in the most severe form of dengue fever (30), and in patients with active tuberculosis (TB), where it is associated an impaired IFNα production (31). Particularly, it was observed a more significant reduction of pDCs both in the pulmonary and extrapulmonary forms of TB, while mDCs count was lower in the extrapulmonary form of TB. It has been showed that DC counts can increase during highly active antiretroviral therapy and normalize after anti-tuberculous therapy in responder patients. Similarly, in our patient DCs subsets progressively increased and normalized after the end of the 18-months therapy. In addition, evaluation of DCs maturation *in vitro* appeared normal, thus suggesting that circulating mDCs and pDCs depletion during the active mycobacterial infection may not be related to abnormal generation of these cell types. Conversely, a disrupted DCs trafficking or increased DCs apoptosis due to the defective IFNγR1-mediated signaling might account for DCs depletion during mycobacterial infection. Further studies will allow to elucidate DCs role in response against mycobacterial disease and evaluate the DC counts as a diagnostic tool to monitor treatment response or to predict development of mycobacterial infection in patients with inborn errors of IFNγ immunity.

## Data Availability Statement

All datasets presented in this study are included in the article/supplementary materials.

## Ethics Statement

The studies involving human participants were reviewed and approved by Comitato Etico Spedali Civili of Brescia. Written informed consent to participate in this study was provided by the participants' legal guardian/next of kin. Written informed consent was obtained from the minor(s)' legal guardian/next of kin for the publication of any potentially identifiable images or data included in this article.

## Author Contributions

LD followed the patient and wrote the manuscript. DV, MG, and NT performed the assays. DM performed the immunophenotyping assay. WV and SL performed the histology study of lymph nodes. LD, DV, and RB analyzed all the data and the results. SG, JB, and J-LC participated in the conception of the work and revised the manuscript. RB designed and supervised the whole study. All authors participated in manuscript writing and/or revised the manuscript and approved the final version.

## Conflict of Interest

The authors declare that the research was conducted in the absence of any commercial or financial relationships that could be construed as a potential conflict of interest.
